# Valproic acid disrupts the biomechanics of late spinal neural tube closure in mouse embryos

**DOI:** 10.1016/j.mod.2017.12.001

**Published:** 2018-02

**Authors:** Amy Hughes, Nicholas D.E. Greene, Andrew J. Copp, Gabriel L. Galea

**Affiliations:** Developmental Biology of Birth Defects, UCL GOS Institute of Child Health, London, UK

**Keywords:** Neural tube, Valproic acid, Mouse, Closure 5, Biomechanics

## Abstract

Failure of neural tube closure in the early embryo causes neural tube defects including spina bifida. Spina bifida lesions predominate in the distal spine, particularly after exposure to the anticonvulsant valproic acid (VPA). How VPA specifically disturbs late stages of neural tube closure is unclear, as neurulation is usually viewed as a uniform ‘zippering’ process along the spine. We recently identified a novel closure site (“Closure 5”) which forms at the caudal extremity of the mouse posterior neuropore (PNP) when completion of closure is imminent. Here we investigated whether distal spina bifida in VPA-exposed embryos involves disruption of Closure 5. Exposure of E8.5 mouse embryos to VPA in whole embryo culture had marked embryotoxic effects, whereas toxic effects were less pronounced in more developmentally advanced (E9) embryos. Only 33% of embryos exposed to VPA from E9 to E10.5 achieved PNP closure (control = 90%). Short-term (8 h) VPA treatment diminished supra-cellular F-actin cables which normally run along the lateral neural folds, and prevented caudal PNP narrowing normally characteristic of Closure 5 formation. Laser ablation of Closure 5 caused rapid neuropore widening. Equivalent ablations of the caudal PNP in VPA treated embryos resulted in significantly less widening, suggesting VPA prevents formation of Closure 5 as a biomechanically active structure. Thus, VPA exposure prevents morphological and biomechanical conversion of the caudal extreme of the PNP during late spinal closure. Closure 5 facilitates neural fold apposition when completion of closure is imminent, such that its disruption in VPA-exposed embryos may lead to distal spina bifida.

## Highlights

• Exposure of mouse embryos to valproate diminishes posterior neuropore closure.
• Valproate treatment prevents caudal neuropore narrowing at late neurulation stages.
• Valproate diminished F-actin cables normally present along the neural folds.
• Laser ablation of the neuropore's caudal extreme normally causes rapid widening.
• Valproate abolishes the caudal neuropore's biomechanical contributions to closure.

## 1 Introduction

Mammalian primary neurulation is a morphogenetic process whereby the flat neural plate bends to form paired neural folds which become medially apposed and fuse at the dorsal midline, forming a closed neural tube (NT) (Nikolopoulou et al., 2017). Fusion begins at specific initiation points, and is then propagated through a zippering process whereby cellular protrusions at the tips of the neural folds reach across the midline to contact the contralateral side (Rolo et al., 2016). Spinal closure initiates at the hindbrain-cervical boundary (Closure 1) and zippers bi-directionally: rostrally to form the cephalic NT and caudally to form the future spine (Nikolopoulou et al., 2017). The open region of spinal NT, referred to as the posterior neuropore (PNP), transitions from a “spade-like” structure at mid-spinal stages to an elliptical shape with a narrowed caudal extreme when completion of closure is imminent (Galea et al., 2017). This shape change is associated with encircling of the PNP by a supra-cellular F-actin ring. We identified cellular protrusions characteristic of active zippering not only at the main site of closure (Rolo et al., 2016) but also at the PNP's caudal canthus in embryos at the final stage of PNP closure. This suggested active caudal-to-rostral as well as rostro-to-caudal closure when spinal neurulation is completed in the low spine. In support of this finding, laser ablation of the caudal canthus resulted in rapid lateral recoil (*i.e.* widening) of the neural folds (Galea et al., 2017). Hence, a new biomechanically active closure point arises at the caudal extremity of the late-stage closing spinal neural tube, which we have denoted “Closure 5” (Galea et al., 2017).

Although Closure 5 has not yet been directly documented in humans, its existence has been inferred from the clustering of spina bifida lesion in the distal lumbo-sacral spine (Van Allen et al., 1993), at which point zippering has progressed unperturbed along most of the embryonic axis. Evidence for this includes the distal spina bifida caused by *in utero* exposure to the anti-epileptic medication valproic acid (VPA) (Robert and Guibaud, 1982, Van Allen et al., 1993). In mice, exposure to VPA during neurulation also impairs NT closure, but the resulting defects primarily affect the cranial region causing exencephaly (the developmental forerunner of anencephaly) (Nau, 1985, Nau and Loscher, 1986). These teratogenic effects are distinct from VPA's anti-epileptic properties as not all of its anti-epileptic metabolites and analogues cause exencephaly when injected into mice (Nau and Loscher, 1986). Caudal spina bifida similar to that seen in humans can be induced in mice by repeated exposure to VPA during mid to late spinal neurulation (three injections on E9) (Ehlers et al., 1992). In cultured rodent embryos, exposure to ~ 1 mM VPA, which is comparable to concentrations measured in the blood of human patients (Suzuki et al., 2011, Vasudev et al., 2001), causes cranial and/or spinal NT defects depending on the treatment regime (Lampen et al., 1999, Seegmiller et al., 1991). However, embryos from certain mouse strains have been reported to be more sensitive to the teratogenic effects of VPA both *in vivo* (Lundberg et al., 2004) and in culture (Naruse et al., 1988). Here we set out to identify a VPA treatment regime which disrupts PNP closure in cultured CD1 mouse embryos and to use this model to determine whether VPA diminishes Closure 5 formation, as a possible explanation for the distal spina bifida in exposed individuals.

## 2 Materials and methods

### 2.1 Embryo culture and treatments

VPA was purchased from Sigma (Cat. No. V0033000) and dissolved with vigorous agitation in neat rat serum. Studies were performed under project license numbers 70/7469 and P8B3095F0 under the UK Animals (Scientific Procedures) Act 1986 and the Medical Research Council's Responsibility in the Use of Animals for Medical Research (1993). Outbred CD1 mice were bred in-house. Embryo culture was performed essentially as previously described by our group (Copp et al., 2000).

For experiments starting at E8.5, mice were mated overnight and the morning a plug was found was considered E0.5. Pregnant females were sacrificed in the morning of E8.5 (~ 8 somites at the start of culture) and their embryos cultured for 24 h.

For experiments starting at E9, mice were mated during the day, and noon of the day a plug was found was considered E0. Pregnant females were sacrificed in the morning of E9 (~ 15 somites at the start of culture) and their embryos cultured for 8 h or 24–36 h as indicated.

At the end of culture, embryos were imaged in their yolk sac using a Leica DFC490 mounted on a Zeiss Stemi SV-11 stereomicroscope, dissected out of their extraembryonic membranes and fixed in 4% PFA. PNP images were then captured using the same setup to analyse PNP dimensions and embryo lateral images were captured to measure dorsal length as a curved line from the otic vesicles to the caudal tip, using Fiji (Schindelin et al., 2012).

### 2.2 Wholemount staining and confocal microscopy

Embryo whole-mount staining with Alexa Fluor™-568 conjugated phalloidin, DAPI and far red CellMask™ was as previously described (Galea et al., 2017). Images were captured on a Zeiss Examiner LSM880 confocal using a 20 ×/NA1.0 Plan Apochromat dipping objective. Embryos were typically imaged with X/Y pixel sizes of 0.59 μm and Z-step of 1.0 μm (speed = 8, bidirectional imaging, 1024 × 1024 pixels). Images were processed with Zen2.3 software and visualised as maximum projections in Fiji.

### 2.3 Laser ablation

Closure 5 laser ablations were performed as previously described using a MaiTai laser (SpectraPhysics Mai Tai eHP DeepSee multiphoton laser, 800 nm wavelength, 100% laser power, 65.94 μs pixel dwell time, 1 iteration). Reflection images of live embryo PNPs were obtained using a 10 ×/NA0.5 Plan Apochromat dipping objective (633 nm laser wavelength). PNPs were imaged before and immediately after ablation, taking approximately 3 min to capture each Z-stack.

### 2.4 Statistical analysis

Comparisons between two groups were by Student's unpaired *t*-test accounting for homogeneity of variance in Excel or in SPSS (IBM Statistics 22). Comparison of multiple groups was by one-way ANOVA with *post-hoc* Bonferroni in SPSS. Linear regression F-test was in OriginPro 2016 (Origin Labs). Multivariate analysis for serial PNP width or change in width measurements were by linear mixed models in SPSS accounting for the fixed effects of treatment and percentage of PNP length in repeated measures from each, with a *post-hoc* Bonferroni. Graphs were made in OriginPro 2016 (Origin Labs) and are represented as box plots or as the mean ± SEM when several groups are shown per measurement level. *p* < 0.05 was considered statistically significant.

## 3 Results

### 3.1 Closure 5 forms when completion of PNP closure is imminent

**Fig. 1:**
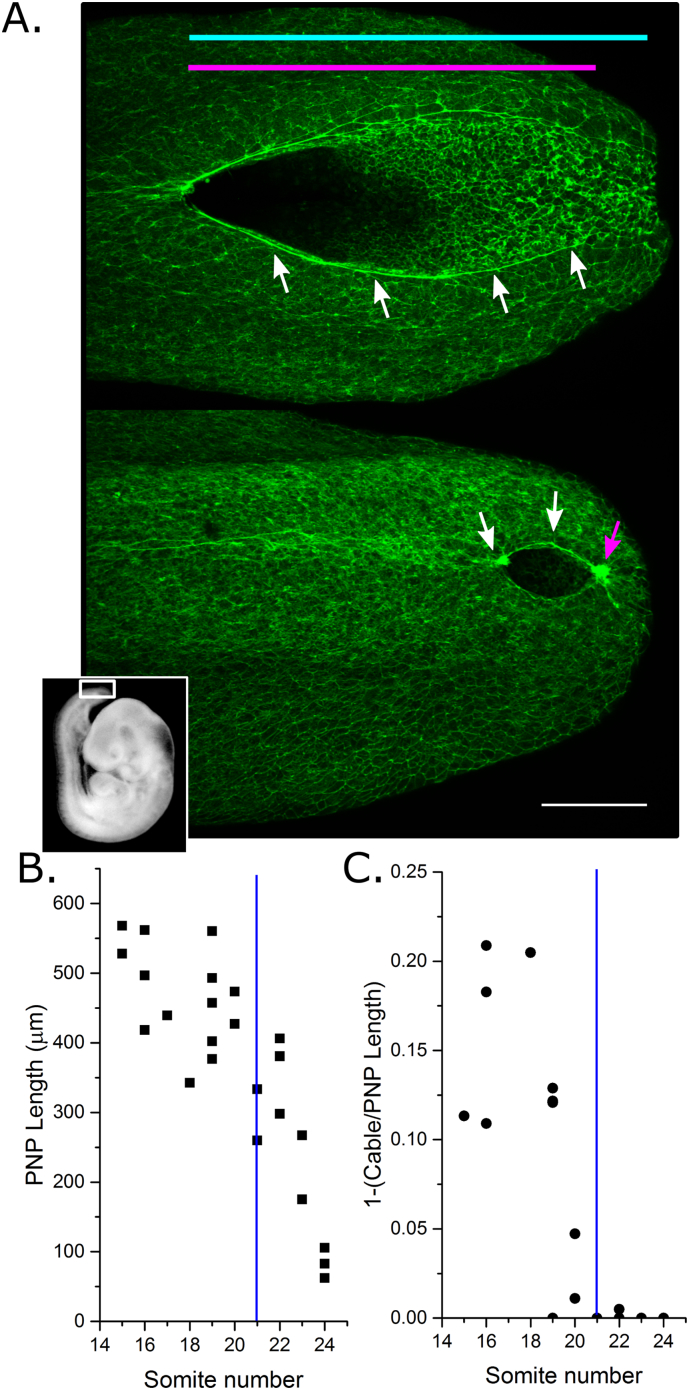
Closure 5 forms when completion of PNP closure is imminent in CD1 mouse embryos. A) Representative dorso-ventral views of whole mount phalloidin-stained PNPs at mid (top) and late (bottom) spinal neurulation stages. The white box in the embryo inset image indicates the location of the PNP. White arrows indicate the F-actin cable along the neural folds. The magenta arrow indicates the position of Closure 5. PNP length (cyan line) and F-actin cable linear length (magenta line) were calculated. Scale bar = 100 μm. B) PNP length decreases with advancing somite stage, with completion of closure occurring ~ 25 somites in CD1 embryos used in this study. C) The proportion of the PNP not encompassed by the F-actin cable decreases until the cable encircles the PNP at ~ 21 somites in CD1 embryos. Vertical blue lines = 21 somites.

The PNP of mouse embryos transitions from a “spade-like” morphology at mid-spinal levels to an elliptical shape when completion of closure is imminent and Closure 5 has formed (Fig. 1A). An F-actin cable is visible running along the neural folds at early stages, and this encircles the PNP at late somite stages (Fig. 1A) (Galea et al., 2017). Consequently, at early stages the F-actin cable does not reach the caudal limit of the PNP, but from the ~ 21 somite stage the cable forms a purse string-like structure around the PNP (Fig. 1B,C and data previously reported (Galea et al., 2017)). The F-actin cable reached the caudal limit of the PNP in 90% (10/11) of embryos with ≥ 21 somites, but only 10% (1/14) of embryos with ≤ 20 somites analysed in the present study.

### 3.2 VPA exposure retards embryonic development and disrupts PNP closure

**Fig. 2:**
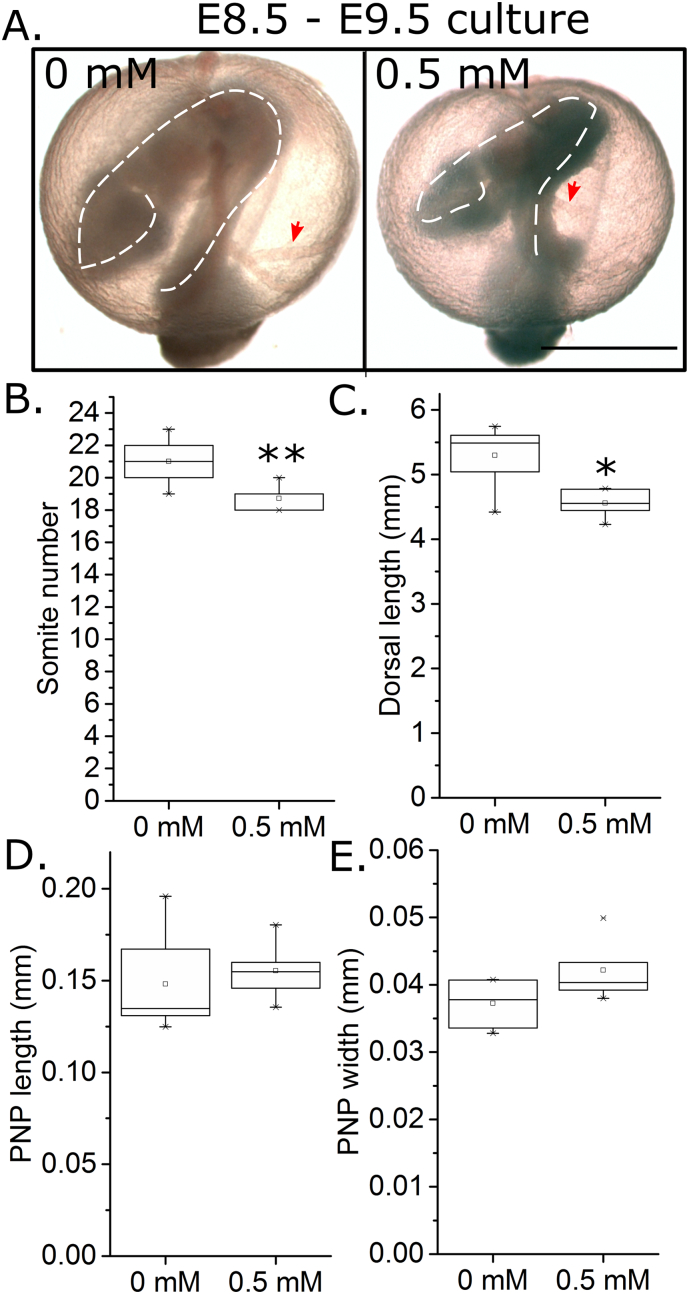
Valproic acid exposure in early neurulation diminishes embryo growth. A) Representative images of CD1 embryos cultured for 24 h from E8.5 to E9.5 in 0 mM or 0.5 mM VPA (1 mM VPA decreased viability at this early developmental stage). The outline of the embryos (white dashed lines) and yolk sac vessels (red arrows) are indicated. Scale bar = 1 mm. B) Somite number was quantified in all cultured embryos at the end of culture and was significantly diminished by 0.5 mM VPA treatment, n = 8 per group. C–E) Comparing embryos which reached 19–22 somite stage at the end of culture (n = 5–6), 0.5 mM VPA treatment significantly diminished dorsal length (C) without altering PNP length (D) or width (E). *p < 0.05, **p < 0.01.

The neuro-teratogenic and embryotoxic effects of VPA vary in different mouse strains (Naruse et al., 1988) and gestation ages (Kao et al., 1981), but culture in ~ 1 mM VPA has previously been reported to cause NTDs in cultured embryos (Kao et al., 1981, Naruse et al., 1988, Seegmiller et al., 1991). In pilot studies, culture of E8.5 CD1 embryos in 1 mM VPA caused clear evidence of embryo toxicity, namely absence of active yolk sac circulation in 7/8 embryos compared with 1/9 vehicle-treated embryos (*X*^2^: *p* = 0.002). All embryos treated with 0.5 mM VPA had visible yolk sac circulation at the end of culture, but treatment delayed embryo development as evidenced by a smaller somite number after 24 h of treatment (Fig. 2A,B) and reduced embryo dorsal length at similar somite stages (Fig. 2C). Despite these clear toxic effects, 0.5 mM VPA did not significantly alter PNP dimensions in embryos which achieved similar somite stages (Fig. 2D,E). Hence, VPA diminishes embryo development during early neurulation, but has no detectable effects on spinal neural tube closure.

**Fig. 3:**
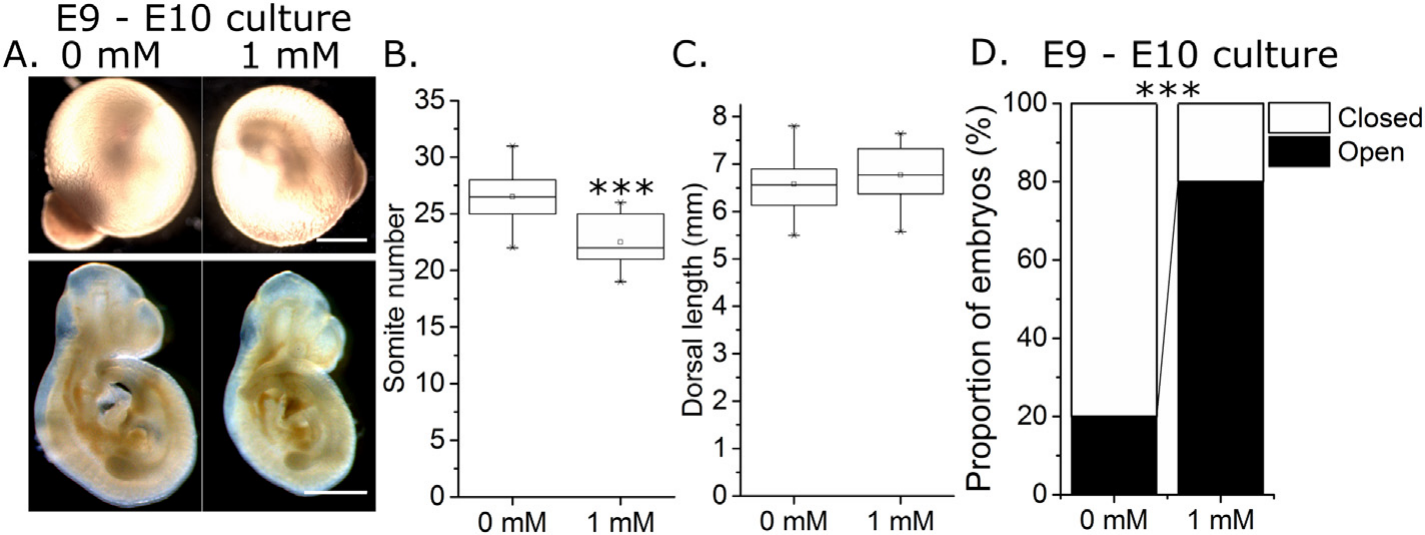
Valproic acid exposure from mid-neurulation diminishes embryo development and chronologically delays posterior neuropore closure. A) Representative images of CD1 embryos cultured for 24 h from E9 to E10 in 0 mM or 1 mM VPA. Embryos are shown in their yolk sacs at the end of culture (highly vascular) and post dissection and fixation. Scale bars = 1 mm. B) Somite number was quantified in all cultured embryos at the end of culture and was significantly diminished by 1 mM VPA treatment, n = 10 per group. C) Dorsal length of 22–26 somite embryos was not significantly different between 0 mM (n = 5) and 1 mM (n = 8) treated embryos. D) A significantly smaller proportion of embryos treated with 1 mM VPA achieved PNP closure by the end of culture period than the more developmentally advanced 0 mM-treated embryos, n = 10 per group. ***p < 0.001.

Embryos were next cultured for 24 h from a later gestational age (E9), and were found to be less sensitive to the effects of VPA: all embryos cultured in 1 mM VPA (which was toxic for E8.5 embryos) had visible yolk sac circulation at the end of culture (Fig. 3A). This treatment delayed somite number increase (Fig. 3A,B), but did not significantly change embryo dorsal length relative to somite stage matched control embryos (Fig. 3C). PNP length and width could not be compared between groups as most control embryos achieved developmental stages > 25 somites and consequently completed PNP closure. A significantly smaller proportion of 1 mM VPA-treated embryos achieved PNP closure within the same time frame (Fig. 3D). However, this comparison is confounded by treated embryos being less developmentally advanced than controls.

**Fig. 4:**
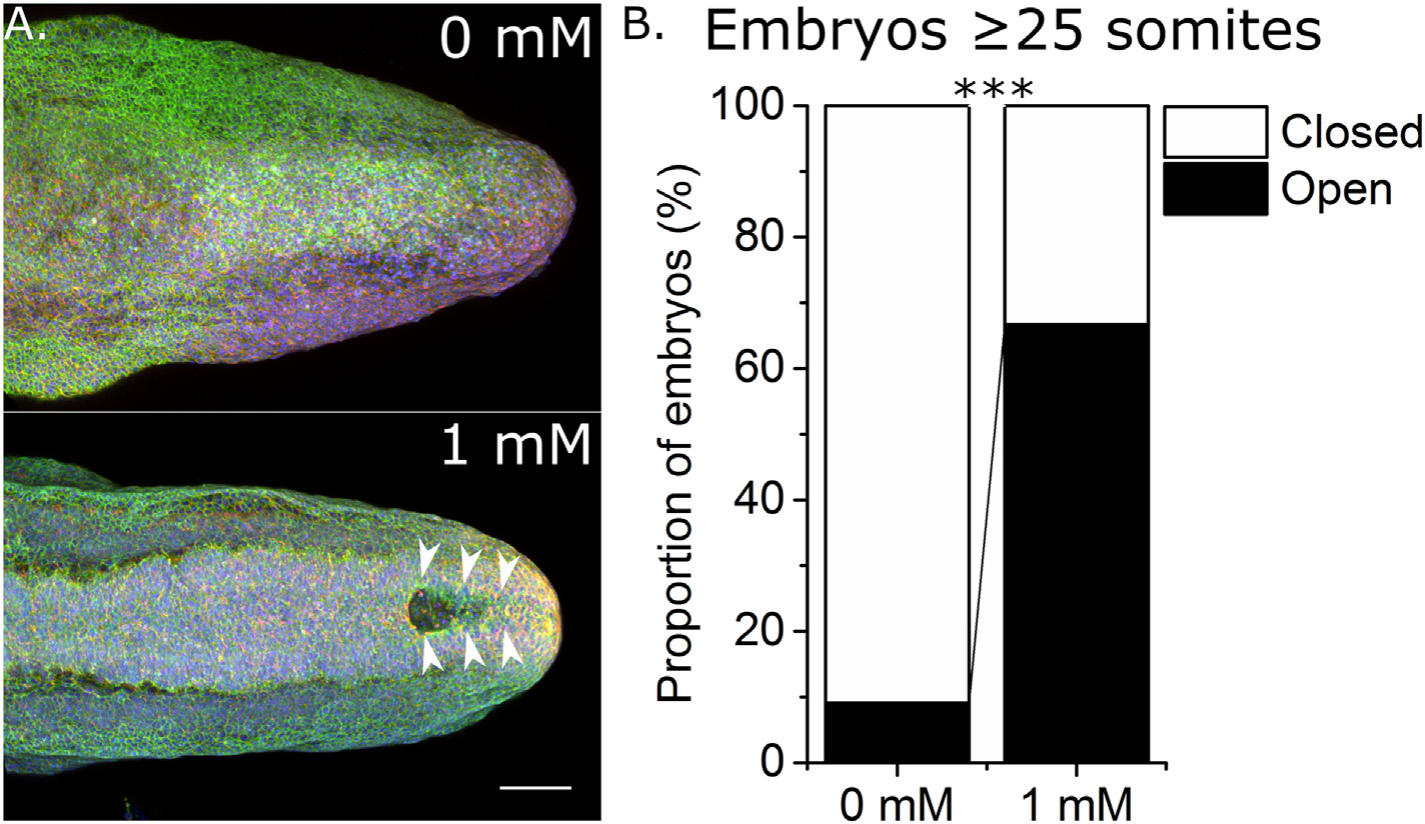
Valproic acid exposure from mid-neurulation disrupts completion of posterior neuropore closure. A) Representative images of whole mount phalloidin (green), cell mask (red) and DAPI (blue) stained caudal regions of 27 somite stage embryos cultured for 24–36 h in 0 mM or 1 mM VPA. Arrows indicate the open PNP in the 1 mM treated embryo. Scale bar = 100 μm. B) Whereas the majority of cultured control (n = 11) embryos at somite stages ≥ 25 achieved PNP closure, a significantly smaller proportion of 1 mM VPA treated embryos (n = 9) with ≥ 25 somites achieved PNP closure. ***p < 0.001.

In order to compare VPA-treated and untreated embryos at similar developmental stages, cultures were extended to 36 h such that the majority of embryos in both treatment groups achieved ≥ 25 somites. Of these, 90% of control embryos achieved PNP closure whereas only 33% of 1 mM VPA treated embryos completed PNP closure (Fig. 4A,B). Hence, VPA disrupts completion of spinal neural tube closure in cultured mouse embryos.

### 3.3 VPA treatment prevents caudal PNP narrowing and Closure 5 formation

**Fig. 5:**
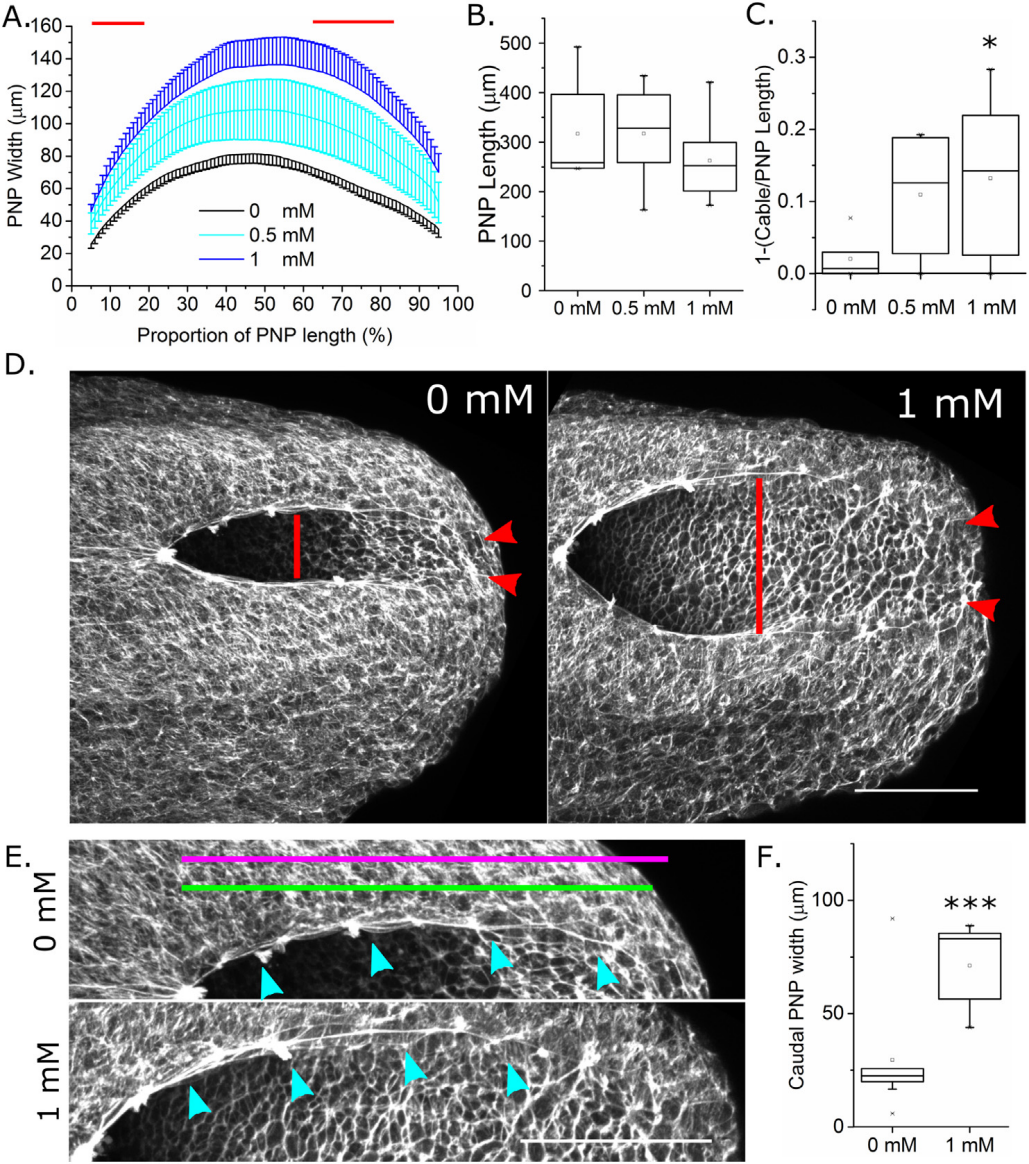
Short-term valproic acid exposure prevents PNP narrowing associated with Closure 5 formation. A–C) 8 h culture in 1 mM VPA significantly increased PNP width (A). This was quantified at every 1% of the PNP's length in a caudal direction from the zippering point at X = 0; red lines indicate significant difference between 1 and 0 mM groups at *p* < 0.05 following Bonferroni correction. PNP length (B) shows a trend towards reduction in the 1 mM group, although this was not significant. C) The proportion of the PNP not occupied by the F-actin cable (quantified as in E) was significantly greater in 1 mM than in control 18–20 somite stage embryos. *p < 0.05; 0 mM, n = 6; 0.5 mM, n = 7; 1 mM, n = 8. D) Representative phalloidin stained whole mount confocal images of embryos treated for 8 h with 0 mM or 1 mM VPA indicating PNP width (red line) and the distance between the neural folds at the caudal margin of the PNP (red arrows). E) Enlarged views of the top neural fold of each embryo in (D). PNP length (magenta line) and linear F-actin cable length (green line) are approximately indicated. The cyan arrows indicate the F-actin cable as it runs along the neural folds. Scale bars = 100 um. F) The distance between the neural folds at the caudal margin of the PNP of 21–24 somite stage embryos cultured for 8 h in 1 mM VPA was significantly greater than those cultured in 0 mM. n = 9 per group, ***p < 0.001.

In order to quantify in greater detail the VPA-associated PNP morphological perturbations, embryos were cultured for 8 h (sufficient time for 4 additional somites to form (Forsberg et al., 1998)) from E9 in 0 mM, 0.5 mM or 1 mM VPA. VPA dose-dependently increased PNP width (Fig. 5A), but not length (Fig. 5B), over this time period. We previously reported that neural fold apposition required for PNP narrowing involves an F-actin cable that biomechanically couples the zippering point to the constricting caudo-ventral PNP (Galea et al., 2017). Although present in all embryos, the F-actin cable length was significantly diminished as a proportion of total PNP length in 1 mM VPA-treated embryos compared with 0 mM controls (Fig. 5C–E). Encircling of the PNP by this F-actin cable is associated with the transition from a spade-like to elliptical morphology with narrowing of the caudal PNP. The width between the neural folds at the caudal margin of the PNP was significantly greater in 1 mM VPA treated embryos than controls (Fig. 5D,F).

**Fig. 6:**
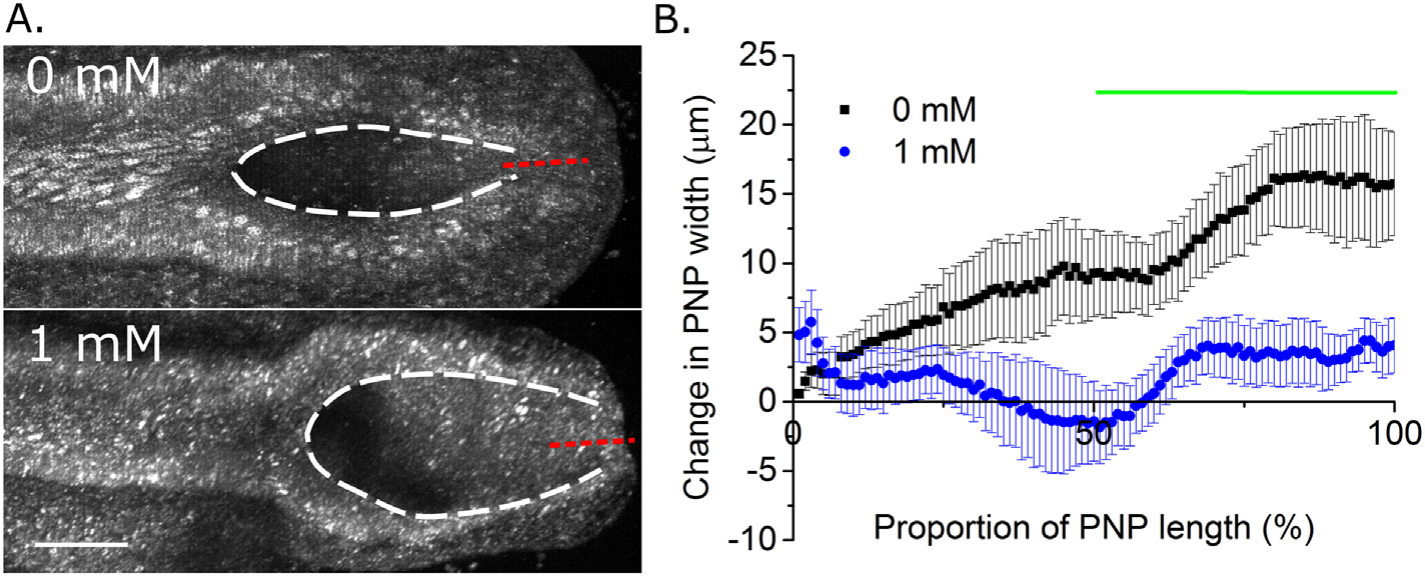
Short-term valproic acid exposure prevents formation of Closure 5 as a biomechanically active structure. A) Representative reflection-mode images of live-imaged 22 somite stage embryos cultured for 8 h in 0 mM or 1 mM VPA. The borders of the neural folds are outlined by the dashed white lines, indicating a more elliptical caudal PNP morphology in the 0 mM embryo. Red dashed lines indicate the Closure 5 laser ablation region. B) The change in PNP width (After–Before) due to Closure 5 laser ablation was quantified at each 1% of the PNP's length as a measure of lateral neural fold recoil. Caudal PNP width increased significantly more in 21–24 somite stage embryos cultured for 8 h in 0 mM (n = 6) than in 1 mM VPA (n = 7). The green line indicates the region of significant difference, *p* < 0.05 following Bonferroni *post-hoc*.

A wider, more spade-like caudal PNP morphology was also visible in live-imaged embryos following 8 h of treatment with VPA, compared with somite stage-matched controls (Fig. 6A). As previously reported in uncultured embryos (Galea et al., 2017), Closure 5 laser ablation resulted in rapid separation of the caudal PNP neural folds in control cultured embryos (Fig. 6B). The equivalent laser ablation of somite stage matched embryos cultured for 8 h in 1 mM VPA resulted in significantly less neural fold separation (Fig. 6B), demonstrating that VPA is responsible for abolishing the biomechanically active closure point at the caudal extremity of the PNP.

## 4 Discussion

The finding of mainly distal spina bifida lesions in human patients exposed to VPA *in utero* led to the suggestion that a terminal PNP closure point, Closure 5, forms at late stages of NT closure (Van Allen et al., 1993). A similar *de novo* caudal closure initiation point (then called the ‘fourth fusion’) was described earlier in the mouse embryo (Sakai, 1989). We recently confirmed in mice that the caudal-most PNP forms a morphologically distinct closure point that is indicated by caudal PNP narrowing into an elliptical shape and encircling of the PNP by an F-actin ring (Galea et al., 2017). Functionally, Closure 5 shows cellular protrusions suggestive of caudal-to-rostral zippering and biomechanically facilitates neural fold apposition (Galea et al., 2017, Sakai, 1989). In the present study we identify an experimental protocol whereby VPA exposure disrupts completion of PNP closure in cultured CD1 mouse embryos and suppresses morphological and biomechanical features of Closure 5 formation.

The growth retarding effects of VPA are well established, as is its ability to cause exencephaly in mice despite primarily predisposing to spina bifida in humans (Kao et al., 1981, Naruse et al., 1988, Seegmiller et al., 1991). As previously reported (Kao et al., 1981), mouse embryos at early stages of neurulation were more sensitive to the toxic effects of 1 mM VPA than more developmentally advanced embryos. This concentration of VPA is clinically relevant given maximum plasma concentrations in human patients can exceed 2 mM (Vasudev et al., 2001), although unlike the human situation cultured embryos are exposed to the same concentration of VPA throughout the culture period without peaks and troughs between dosing intervals. Despite this, VPA exposure at a concentration which effectively diminished development at early to mid-spinal neurulation stages had minimal effects on PNP dimensions, indicating zippering progressed unperturbed from the initiation of culture. Given the spinal level of spina bifida lesions reflects the embryological level at which zippering halts, this is consistent with VPA exposure causing distal but not more proximal spina bifida in humans (Robert and Guibaud, 1982, Van Allen et al., 1993). Similarly, in mice *in vivo*, repeated injection of VPA from E9 also causes distal spina bifida (Ehlers et al., 1992).

The cellular and biomechanical mechanisms required for early/mid and late spinal neurulation are distinct. For example, fusion of the neural folds at early neurulation stages involves Cdc42-dependent filopodial-type zippering protrusions, whereas late stages are typified by broader Rac-dependent membrane ruffles (Rolo et al., 2016). Neural fold bending also changes as the ventral median hinge point, which is present at early and mid-spinal neurulation stages, is absent at late neurulation stages (Shum and Copp, 1996). Another difference is the biomechanical function of the caudal-most PNP, which facilitates neural fold apposition only at late stages when Closure 5 has formed (Galea et al., 2017). Here we provide three lines of evidence that exposure to 1 mM VPA disrupts Closure 5 formation: in treated embryos i) the F-actin cable which encircles the PNP when Closure 5 has formed is significantly diminished, ii) the caudal PNP does not narrow to form an elliptical shape to the same extent as in control embryos, and iii) the caudal PNP minimally influences neural fold apposition as demonstrated by laser ablation, compared with somite stage-matched controls. These effects, as well as increased PNP width, are all evident in embryos treated for 8 h.

Numerous mechanisms have been proposed by which VPA may disrupt NT closure. These include epigenetic dysregulation (Tung and Winn, 2010), inhibition of folate metabolism (Fathe et al., 2014, Roy et al., 2008, Semmler et al., 2017), suppression of nitric oxide signalling (Tiboni et al., 2013, Tiboni and Ponzano, 2015) and increased reactive oxygen species production (Akimova et al., 2017, Tung and Winn, 2011). In addition, VPA's anti-acetylation effects may disrupt post-translational modifications of other effector proteins including those involved in actin regulation. For example, acetylation of the guanine exchanger RhoGDIα diminishes Rho signalling (Kuhlmann et al., 2016) and myosin heavy chain acetylation increases contractility in cardiac muscle (Samant et al., 2015). VPA treatment *in vivo* is associated with increased neuronal actin phosphorylation (Corena-McLeod et al., 2013) and *in vitro* it has been shown to alter actin dynamics leading to increased spreading of mouse fibroblastic cells (Walmod et al., 1999). In the present study, VPA diminished the F-actin cable which normally biomechanically couples the rostral zippering point to the caudal PNP and, eventually, to Closure 5. A potential mechanism underlying this observation is primary disruption of actin turnover, although actin staining appeared minimally affected overall and a truncated cable was visible in VPA-treated embryos. Alternative mechanisms include changes in the function of caudal PNP cells which normally form Closure 5, and changes in the tissue-level biomechanics of the caudal PNP to which the F-actin cable is a contributor.

PNP biomechanical differences between control and VPA-treated embryos are documented here through caudal PNP laser ablation. We had previously used laser ablation to compare biomechanical contributions of the caudal-most PNP to neural fold apposition between mid-spinal neurulation stages, when the PNP is long and Closure 5 has not formed, *versus* late neurulation stages, when the PNP is shorter and Closure 5 has formed (Galea et al., 2017). The laser ablation experiments in the present study were performed in PNPs of similar lengths with *versus* without morphology suggestive of Closure 5, showing that the presence of Closure 5 is required for biomechanical facilitation of neural fold apposition. The lack of PNP widening in VPA-treated embryos following caudal PNP ablation may reflect the absence of medially-apposing force generation, increased stresses within laterally tethering structures precluding caudal neural fold apposition, and/or changes in the material properties of the tissues.

The finding that disruption of Closure 5 is associated with failure of PNP closure in this model, together with its formation of zippering protrusions and biomechanical facilitation of neural fold apposition (Galea et al., 2017), strongly suggest that the caudal closure initiation site is not a “passive player” in the completion of primary neurulation. This is not to say it is invariably critical for closure. For example, Closure 2 in the cephalic region is dispensable for cranial neural fold closure in mice, but its absence greatly increases the risk of exencephaly (Macdonald et al., 1989). Nonetheless, if Closure 5 failure predisposes to spina bifida in humans, delineating the cellular and genetic determinants of its formation may lead to novel approaches to prevent these defects.

Thus, short-term exposure of mouse embryos to VPA from mid-spinal neurulation diminishes Closure 5 as a morphologically distinct and biomechanically active structure. This delays or prevents completion of PNP closure. Identification of the specific mechanisms or developmental windows during which VPA exposure disrupts completion of PNP closure may lead to treatment strategies which decrease the risk of causing spina bifida in human patients.
